# The *Toxoplasma gondii* effector GRA83 modulates the host’s innate immune response to regulate parasite infection

**DOI:** 10.1128/msphere.00263-23

**Published:** 2023-09-28

**Authors:** Amara C. Thind, Caroline M. Mota, Ana Paula N. Gonçalves, Jihui Sha, James A. Wohlschlegel, Tiago W. P. Mineo, Peter J. Bradley

**Affiliations:** 1 Department of Microbiology, Immunology, and Molecular Genetics, University of California, Los Angeles, Los Angeles, California, USA; 2 Molecular Biology Institute, University of California, Los Angeles, Los Angeles, California, USA; 3 Laboratory of Immunoparasitology “Dr. Mário Endsfeldz Camargo,” Institute of Biomedical Sciences, Federal University of Uberlândia, Uberlândia, Minas Gerais, Brazil; 4 Department of Biological Chemistry, David Geffen School of Medicine, University of California, Los Angeles, Los Angeles, California, USA; University of Georgia, Athens, Georgia, USA

**Keywords:** *Toxoplasma gondii*, dense granules, GRA83, GRA15, NF-κB, innate immunity

## Abstract

*Toxoplasma gondii*’s propensity to infect its host and cause disease is highly dependent on its ability to modulate host cell functions. One of the strategies the parasite uses to accomplish this is via the export of effector proteins from the secretory dense granules. Dense granule (GRA) proteins are known to play roles in nutrient acquisition, host cell cycle manipulation, and immune regulation. Here, we characterize a novel dense granule protein named GRA83, which localizes to the parasitophorous vacuole (PV) in tachyzoites and bradyzoites. Disruption of *GRA83* results in increased virulence, weight loss, and parasitemia during the acute infection, as well as a marked increase in the cyst burden during the chronic infection. This increased parasitemia was associated with an accumulation of inflammatory infiltrates in tissues in both acute and chronic infections. Murine macrophages infected with ∆*gra83* tachyzoites produced less interleukin-12 (IL-12) *in vitro*, which was confirmed with reduced IL-12 and interferon-gamma *in vivo*. This dysregulation of cytokines correlates with reduced nuclear translocation of the p65 subunit of the nuclear factor-κB (NF-κB) complex. While GRA15 similarly regulates NF-κB, infection with ∆*gra83/*∆*gra15* parasites did not further reduce p65 translocation to the host cell nucleus, suggesting these GRAs function in converging pathways. We also used proximity labeling experiments to reveal candidate GRA83 interacting *T. gondii*-derived partners. Taken together, this work reveals a novel effector that stimulates the innate immune response, enabling the host to limit the parasite burden.

**Importance:**

*Toxoplasma gondii* poses a significant public health concern as it is recognized as one of the leading foodborne pathogens in the United States. Infection with the parasite can cause congenital defects in neonates, life-threatening complications in immunosuppressed patients, and ocular disease. Specialized secretory organelles, including the dense granules, play an important role in the parasite’s ability to efficiently invade and regulate components of the host’s infection response machinery to limit parasite clearance and establish an acute infection. *Toxoplasma’*s ability to avoid early clearance, while also successfully infecting the host long enough to establish a persistent chronic infection, is crucial in allowing for its transmission to a new host. While multiple GRAs directly modulate host signaling pathways, they do so in various ways highlighting the parasite’s diverse arsenal of effectors that govern infection. Understanding how parasite-derived effectors harness host functions to evade defenses yet ensure a robust infection is important for understanding the complexity of the pathogen’s tightly regulated infection. In this study, we characterize a novel secreted protein named GRA83 that stimulates the host cell’s response to limit infection.

## INTRODUCTION

*Toxoplasma gondii* is an obligatory intracellular parasite of the phylum Apicomplexa that infects a wide variety of avian and mammalian organisms, including humans (1). This widespread parasite is estimated to infect one-quarter of the U.S. population and as many as two billion individuals worldwide (2, 3). Although infection in immunocompetent individuals is typically asymptomatic, infection with the parasite can cause congenital defects in neonates, life-threatening complications in immunosuppressed patients, and ocular disease (4 – 7). After ingesting *T. gondii*, the acute infection is established by rapidly replicating tachyzoites that disseminate from the intestines to peripheral organs, including the central nervous system. This form of the parasite is ultimately controlled by the host’s immune system, but some of the parasites are able to switch to bradyzoites and form latent tissue cysts (8). These cysts persist for the life of the host and can then reactivate the infection during immunosuppressive conditions (4).

To control the infection by *T. gondii*, the host unleashes a wide array of innate and adaptive immune responses (9). These responses involve distinct cellular and humoral mechanisms aimed at dampening the parasite’s rapid replication, thereby avoiding infection-induced pathogenesis. The paramount immune factor against *T. gondii* is interferon-gamma (IFN-γ), which orchestrates effector mechanisms that will successfully kill the parasite during the acute phase of the infection, as well as keep the latent stages quiescent (9 – 12).

*T. gondii’s* initial interactions with innate immune cells play a crucial role in determining the outcome of infection in the murine model (9, 13). The recognition of profilin, a protein associated with the parasites’ actin polymerization, by Toll-like receptors TLR11 and TLR12 located inside the endolysosomes, leads to macrophage production of interleukin 12 (IL-12) (14 – 16). IL-12 production is mediated by nuclear factor-κB (NF-κB), which is the key inducer of IFN-γ by natural killer, TCD4^+^ and TCD8^+^ cells (17 – 19). IFN-γ is readily consumed and activates residing macrophages that are responsible for parasite killing through nitric oxide production (20), tryptophan metabolism (21), and GTPase disruption of the PV (22), among other mechanisms.

*T. gondii*’s ability to successfully invade and replicate within virtually any nucleated cell is dependent on a sequential release of parasite effectors from specialized secretory organelles called the rhoptries and dense granules (23). *T. gondii* tachyzoites actively invade the host cell and replicate within a host-membrane-derived PV, which is able to escape host clearance, enabling intracellular survival. Upon host cell invasion, *T. gondii* secretes an array of dense granule (GRA) proteins into the PV. Some of these GRA proteins regulate vacuolar functions, such as maturation of the PV and nutrient acquisition (24). Other GRAs traverse the vacuolar membrane to reach the host cytoplasm where they actively modulate host cell functions to optimize the parasite’s replication and succeed in colonizing host tissue (23).

Numerous GRAs have been studied in the context of acute infection that contribute to the intricacy of the parasite and host interplay. Some of these GRAs restrict parasite replication and dissemination to limit the infection, while others allow for its persistence in different host tissues. One of these is GRA16, which binds two host enzymes, the deubiquitinase HUASP and PP2A phosphatase, resulting in the accumulation of host c-Myc, regulation of p53, and the cell cycle, which ultimately results in attenuated virulence (25, 26). Another of these effectors is GRA28, which is responsible for facilitating parasite dissemination during pregnancy by inducing CCL22, a chemokine critical for immune tolerance during pregnancy in placental and monocyte-like cells (27). GRA28 is also the mediator of upregulated CCR7 and responsible for the hypermigratory phenotype exhibited by *T. gondii*-infected macrophages (28). While these GRAs serve to enable infection, GRA24 dampens parasite burden by interacting with host p38α MAPK, leading to its activation and downstream stimulation of inflammatory cytokines (29). Similarly, GRA15 interacts with host tumor necrosis factor receptor-associated factors (TRAFs) and activates the NF-κB pathway to drive transcription of IL-12 (30 – 32). These are just some of the examples of GRAs that highlight the parasite’s diverse arsenal of effectors that modulate infection. Elucidating the full complement of the parasite-derived effectors that hijack host functions to favor intracellular survival, as well as those that regulate host defense, is important for understanding the intricacy of how the infection is regulated. A mechanistic understanding of the processes is also important for the establishment of efficient prophylactic and therapeutic protocols to effectively treat *T. gondii* infections.

Herein, we characterize a new GRA protein named GRA83 that is secreted into the vacuole in both tachyzoites and bradyzoites. Disruption of *GRA83* surprisingly increases parasite burden and overall susceptibility to *T. gondii* infection in mice. This increased virulence correlates with the dysregulation of cytokines in acutely infected mice, indicating that GRA83 modulates innate immune mechanisms. This dysregulation of cytokines is linked to a decrease in NF-κB activation in ∆*gra83* parasite infections. Removal of GRA83 also results in a substantial increase in cyst burden observed in chronically infected mice. We also report potential *T. gondii*-derived GRA83 interacting partners through proximity labeling using GRA83 as bait. Together, we determined that GRA83 enhances the host response to *Toxoplasma* infection and, therefore, plays an important role in the interplay between the parasite and its host.

## RESULTS

### GRA83 is a dense granule protein that is secreted into the PV

**Fig 1:**
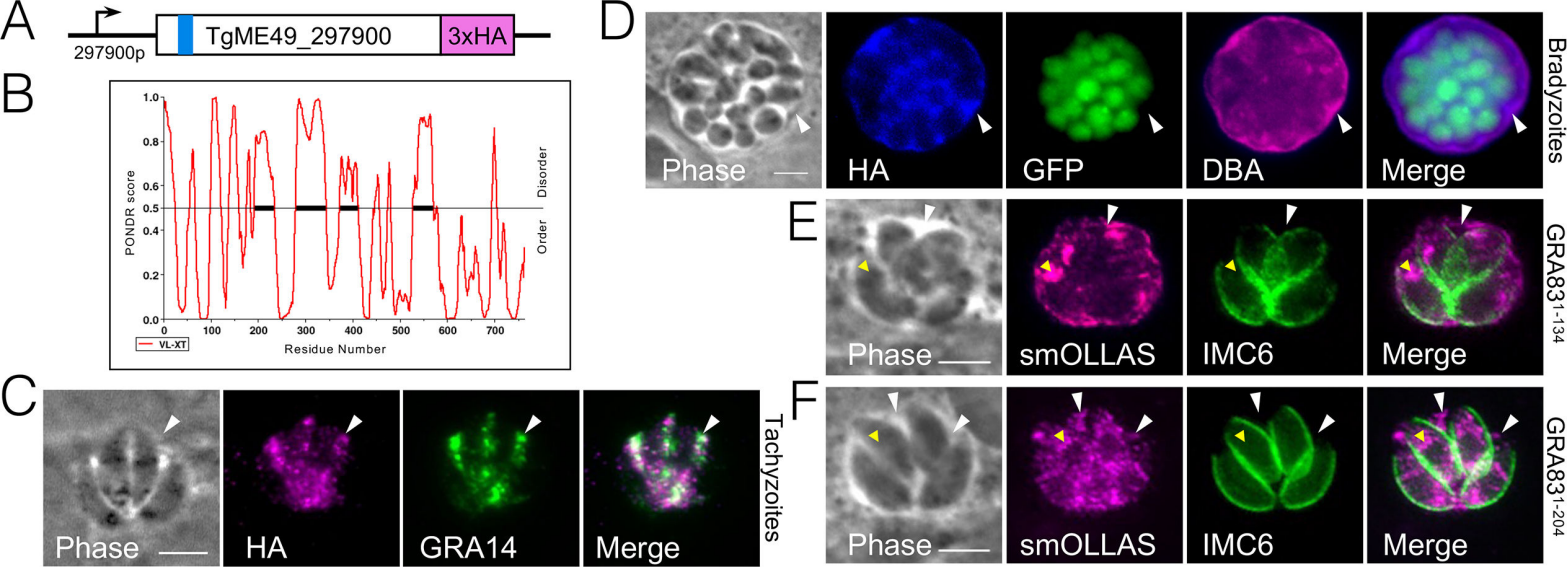
TGME49_297900 is a dense granule protein that localizes to the PV. (**A**) Diagram of TGME49_297900 showing its N-terminal hydrophobic domain (blue bar), as well as the C-terminal endogenous 3xHA epitope tag. (**B**) GRA83 is 40% disordered overall, as determined by the Predictor of Natural Disordered Regions (PONDR) algorithm. Scores higher than 0.5 are considered disordered. (**C**) IFA of GRA83^3xHA^ parasites demonstrating protein accumulation in the vacuole between the tachyzoites (arrowhead) and colocalization with known dense granule protein GRA14. Magenta: anti-HA; green: anti-GRA14. (**D**) IFA of GRA83^3xHA^ *in vitro* switched bradyzoites shows robust staining of GRA83 in the bradyzoite vacuole (arrowhead). The parasites express cytosolic GFP confirming bradyzoite differentiation. DBA stains lectins present on the cyst wall. Images were taken after 3 days of growth in alkaline-stressed conditions. Blue: anti-HA; magenta: DBA. (**E and F**) IFA of GRA83 endogenously smOLLAS-tagged after residues 134 or 204. The truncated protein is secreted into the PV (white arrowhead), while some protein is also present in the parasite (yellow arrowhead). Magenta: anti-OLLAS; green: anti-IMC6. All scale bars are = 5 µm.

TGME49_297900 was initially identified in our MAG1-BioID studies, which involved using *in vivo* biotinylation of the bradyzoite PV to discover novel dense granule proteins (33). TGME49_297900 was also predicted to be a GRA protein by the recent *T. gondii* hyperLOPIT (localization of organelle proteins by isotope tagging) analysis (34). OrthoMCL predicts that TGME49_297900 is restricted to *Toxoplasma* and its closest relatives (*Neospora, Hammondia,* and *Besnoitia*), but is not present in other coccidians or apicomplexans (35). TGME49_297900 encodes an ~83 kDa protein that lacks a signal peptide but contains a predicted hydrophobic domain (residues 74–92) near the N-terminus, which could play a role in secretion or membrane association (Fig. 1A) (36). While many GRAs are polymorphic between the three major strains of *T. gondii*, there are only three amino acid changes in GRA83 between type I and type II/III strains. Similar to other GRAs, TGME49_297900 contains intrinsically disordered regions and has no discernible protein motifs or domains that suggest its function (Fig. 1B). In agreement with this, AlphaFold provides only a very low confidence structural prediction for TGME49_297900 (Fig. S1) (37, 38).

To confirm the localization of the protein, we used endogenous gene tagging to add a C-terminal 3xHA epitope tag to TGME49_297900 in Prugniaud∆*ku8*0∆*hxgprt* parasites, which also express GFP under the control of the bradyzoite specific *LDH2* promoter (39, 40) (Fig. 1A). Immunofluorescence assays (IFA) show that the tagged protein traffics to the PV lumen and colocalizes with GRA14 (41) in tachyzoites and thus we named it GRA83 (Fig. 1C). Because we found this GRA in our previous data set that is enriched for bradyzoite proteins and its expression is upregulated in bradyzoites, we differentiated the tagged parasites to bradyzoites *in vitro* (42). Successful transition from the tachyzoite to the bradyzoite stage was confirmed by the expression of GFP and with Dolichos biflorus agglutinin (DBA) staining, which recognizes N-acetylgalactosamine on the bradyzoite cyst wall protein CST1 (43). IFA of the bradyzoites demonstrated that GRA83^3xHA^ also localizes to the PV in this stage of the parasite (Fig. 1D). We also tested whether the N-terminal region containing the hydrophobic domain is sufficient for secretion into the vacuole by integrating a smOLLAS (spaghetti monster *Escherichia coli* OmpF linker and mouse langerin fusion sequence) tag after sequences encoding the first 134 or 204 amino acids of the protein (44). IFA of these parasites showed that the N-terminal region can direct secretion into the PV (Fig. 1E and F) although some material also remained within the parasite, likely arrested in the secretory pathway as it partially colocalizes with the endoplasmic reticulum (ER) marker SERCA (45) (Fig. S2A and B). This is likely due to misfolding of the smOLLAS tag fusions, which may partially disrupt trafficking, but it is also possible that more C-terminal regions of the protein play a role in secretion. Together, these data demonstrate that TGME49_297900 is a GRA protein that is secreted into the PV in both tachyzoites and bradyzoites and that the N-terminal region containing the hydrophobic domain functions in secretion.

### GRA83 does not affect parasite fitness *in vitro*

**Fig 2:**
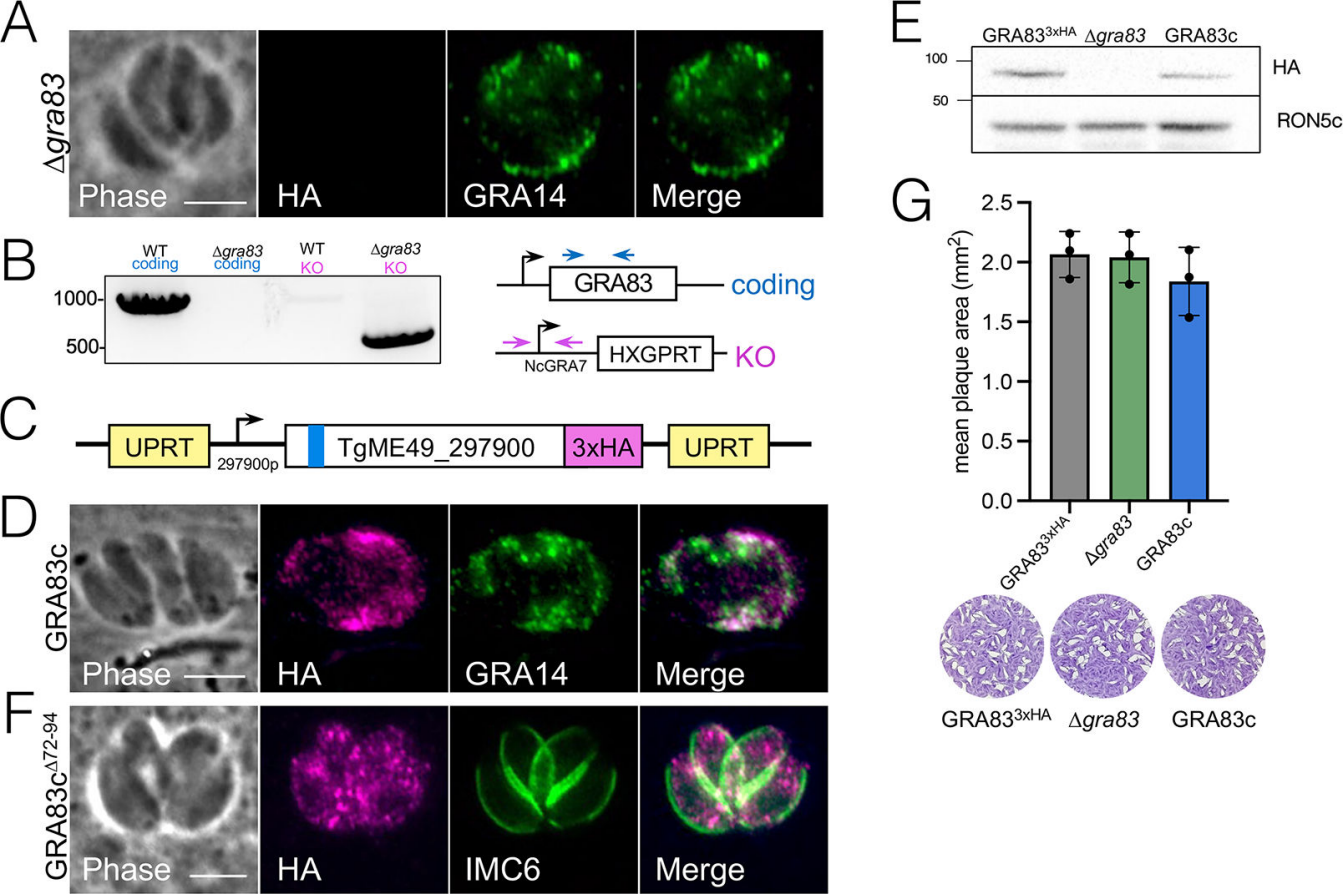
GRA83 is not required for growth in HFFs *in vitro*. (**A**) IFA of ∆*gra83* parasites shows the absence of GRA83^3xHA^. Magenta: anti-HA;green: anti-GRA14. (**B**) PCR verification shows that the Δ*gra83* strain contains the correct amplicon for the replacement of *GRA83* with the selectable marker *HXGPRT* and lacks the *GRA83*-coding amplicon. The diagram illustrates the positions of primers used to amplify the *GRA83* coding sequence (blue arrows) and the knockout construct in the *GRA83* locus (magenta arrows). (**C**) Construct for GRA83 complementation showing the full-length GRA83 tagged with 3xHA driven by its endogenous promoter and targeted to the *UPRT* locus. (**D**) IFA of GRA83-complemented parasites (GRA83c) shows restoration of GRA83 localization in the PV. Magenta: anti-HA; green: anti-GRA14. (**E**) Western blot of GRA83^3xHA^, ∆*gra83,* and GRA83c parasites shows the absence of endogenous GRA83 in the knockout strain and approximate levels restored in the GRA83c strain. GRA83 was detected with anti-HA; RON5c was used as a loading control and detected with anti-RON5c. (**F**) IFA of GRA83c^∆72-94^ parasites shows that the deletion of the predicted hydrophobic region (residues 72–94) results in cytosolic localization of GRA83. Magenta: anti-HA; green: anti-IMC6. (**G**) Quantification of plaque assays depicts no *in vitro* replication defect of ∆*gra83* parasites cultured in HFFs. The mean of each triplicate experiment per strain is shown. No significance was detected using one-way ANOVA test. Representative images of the stained plaques are shown below the quantification. Scale bars = 5 µm.

To assess the function of GRA83, we disrupted its gene from the 3xHA tagged background using CRISPR/Cas9 to obtain ∆*gra83* parasites. The deletion was confirmed by the lack of staining of the tagged protein by IFA and PCR verification (Fig. 2A and B). We then complemented the knockout with a full-length, 3xHA-tagged GRA83 construct driven by its endogenous promoter, which was targeted to the uracil phosphoribosyl transferase (UPRT) locus (Fig. 2C) (46). The resulting complemented strain was named GRA83c. IFA and western blot analyses showed that complementation restored GRA83 localization to the PV and approximate expression levels of the protein (Fig. 2D and E). To test whether the N-terminal hydrophobic region of GRA83 is important for the secretion into the vacuole, we deleted residues 72–94 in the full-length complementation construct and expressed it in the ∆*gra83* strain. We found that removal of the hydrophobic region restricts GRA83 within the parasite with no secreted protein detected in the PV (Fig. 2F). We then evaluated the replication of ∆*gra83* parasites by plaque assay and found no significant difference in plaque size between GRA83^3xHA^, ∆*gra83,* and GRA83c strains, demonstrating that this protein does not contribute substantially to parasite fitness *in vitro* in human foreskin fibroblasts (HFFs) (Fig. 2G).

### GRA83 potentiates mouse survival and parasite restriction *in vivo*

**Fig 3:**
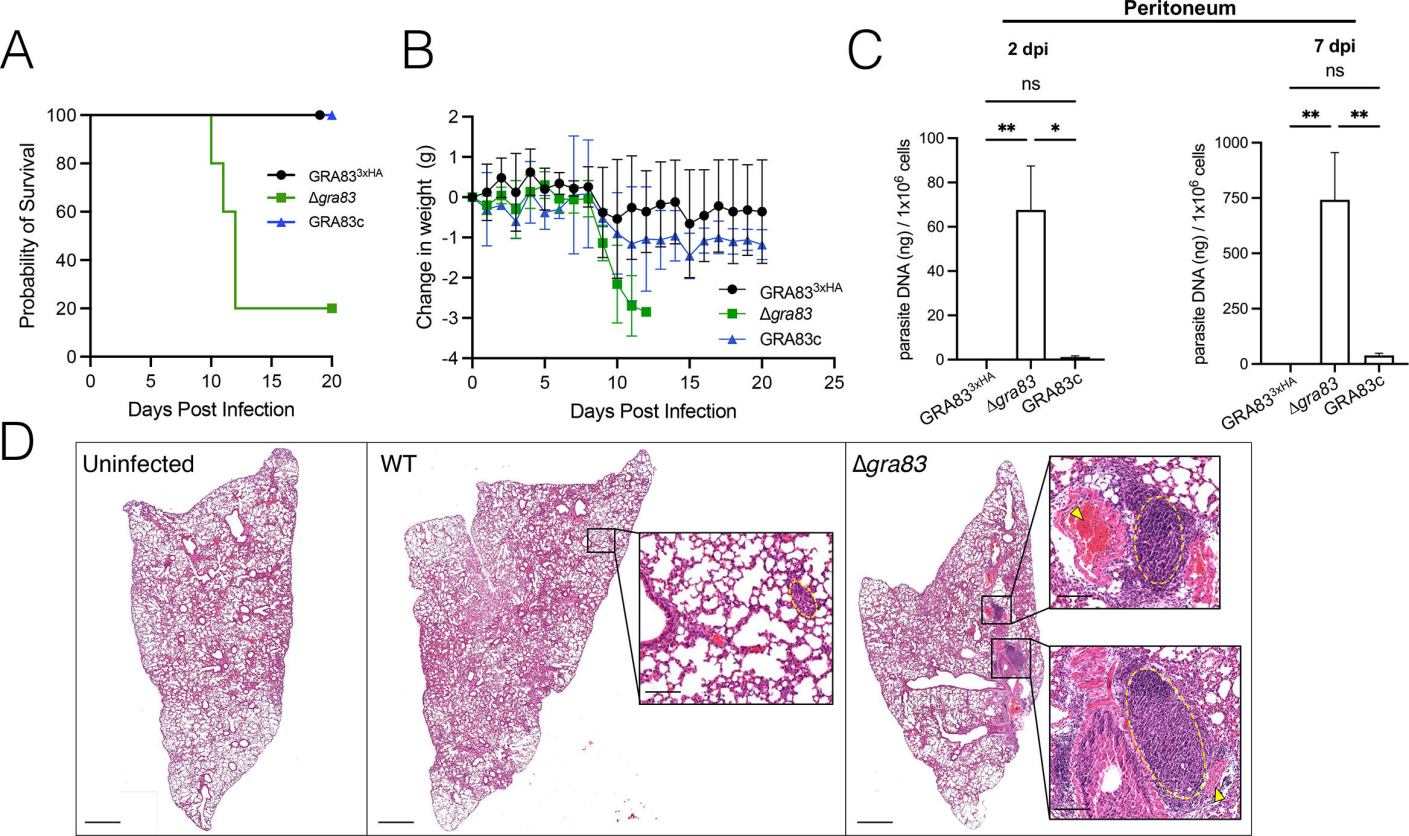
Deletion of *GRA83* increases virulence, parasitemia, and inflammation during the acute infection. (**A**) C57BL/6 mouse survival assay demonstrates the increased virulence of ∆*gra83* parasites (*n* = 5 mice per strain). Mice were intraperitoneally infected with 10^3^ parasites each. Differences between groups were compared using Kaplan Meier survival analysis, through log-rank (Mantel-Cox) test (*P <* 0.015). (**B**) Weight change of C57BL/6 mice from the virulence assay shown in panel A. Results are shown as the average weight change per group of infected mice each day. (**C**) C57BL/6 mice infected with 300 parasites were evaluated for parasite burden by qPCR. Peritoneal cells were evaluated for the number of copies of the repetitive 529 bp DNA fragment in *T. gondii* (*n* = 5 mice/strain, with two technical replicates each). Results are expressed as mean ± SEM. Data were analyzed using Kruskal-Wallis one-way ANOVA test; *P < 0.05; **P < 0.005. (**D**) Images of hematoxylin and eosin (H&E) stained lung sections of uninfected or infected C57BL/6 mice with 300 tachyzoites of the GRA83^3xHA^ and ∆*gra83* strains at 7 dpi. Scale bars = 1 mm. Insets show the magnification of the yellow circled regions highlighting inflammatory infiltrates or BALT hyperplasia. Arrows show the migration of mononuclear cells through surrounding blood vessels. Scale bars = 100 µm.

To determine whether GRA83 is relevant in the context of an *in vivo* infection, we assessed the virulence of the GRA83^3xHA^, ∆*gra83*, and GRA83c parasites in a C57BL/6 mouse survival assay. Surprisingly, we found that the ∆*gra83* strain was significantly more virulent, as most of the mice from this group succumbed to the infection at 10–12 days post-infection (dpi), while all of the mice infected with the GRA83^3xHA^ or GRA83c lines survived the experimental period (Fig. 3A). Accordingly, mice infected with the knockout strain also showed increased weight loss (Fig. 3B), indicating a more severe illness during the acute phase of the infection.

To further explore this phenotype, we assessed parasite burden by qPCR at 2 and 7 dpi (Fig. 3C). In agreement with the enhanced virulence, mice infected with ∆*gra83* parasites had a significantly higher parasite burden in the peritoneum, with levels several fold greater than the GRA83^3xHA^ strain at both time points, and this increased burden was reversed by complementation. We next sought to determine if the increased virulence of the ∆*gra83* parasites led to inflammatory lesions in the nearby lung tissue, one of the preferred targets of parasite replication during the acute phase (47). For mice infected with the wild-type control strain, we observed interstitial inflammatory infiltrates throughout the parenchyma of the lungs at 7 dpi, composed mainly of mononuclear cells (Fig. 3D). On the other hand, histological analysis of the lungs of ∆*gra83*-infected mice showed compromised tissue integrity due to the extensive interstitial inflammation associated with sites of bronchus-associated lymphoid tissue (BALT) hyperplasia. Together, these data demonstrate that the loss of GRA83 results in enhanced growth of the parasites in the acute phase of the infection.

### GRA83 impacts cyst burden and inflammation in the brains of chronically infected mice

**Fig 4:**
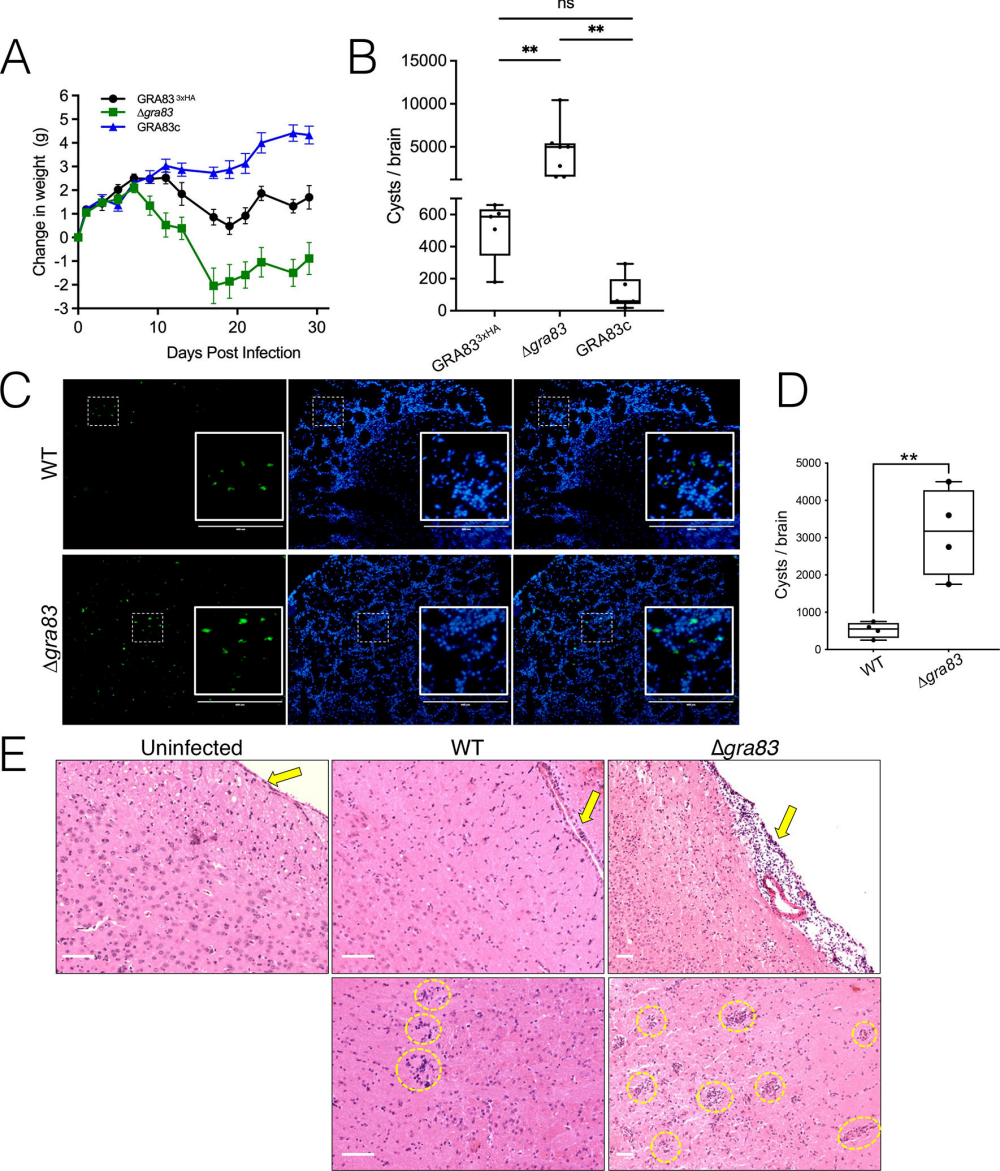
Deletion of *GRA83* increases cyst burden and brain inflammation during the chronic infection. (**A**) Weight change of CBA/J mice infected with 400 parasites of each strain. Results are shown as the average weight change per group of infected mice each day (GRA83^3xHA^: *n* = 5, ∆*gra83*: *n* = 7, and GRA83c: *n* = 6). (**B**) Brain cyst burden of CBA/J mice at 30 dpi infected with 400 parasites from each of the indicated strains as in panel A. Results are shown as values with min and max whiskers. One-way ANOVA test; **P < 0.01. (**C**) Images of frozen brain sections of infected C57BL/6 mice at 32 dpi with 300 tachyzoites each. Cysts are *LDH2*-GFP^+^ and 4′,6-diamidino-2-phenylindole (DAPI) labels the cell nuclei. Insets show the magnification of the boxed regions highlighting the cysts. Scale bars = 400 µm. (**D**) Quantification of brain cyst numbers from panel C (*n* = 4 per group). Results are shown as values with minimum and maximum whiskers. Two-tailed *t*-test, **P < 0.05. (**E**) Images of H&E-stained brain sections of uninfected or infected C57BL/6 mice with 300 tachyzoites of the GRA83^3xHA^ or ∆*gra83* strains at 30 dpi. Inflammatory infiltrates containing mononuclear cells in the brain parenchyma or around blood vessels are circled in yellow. Arrows point at the meninges of samples analyzed from the different groups showing an intense gathering of mononuclear cells indicating meningitis in mice infected with the ∆*gra83* strain. Scale bars = 50 µm.

To assess whether GRA83 is important for the chronic infection, we first infected CBA/J mice, which produce higher cyst numbers (48), with sublethal doses of tachyzoites to assess weight loss and cyst burden. As observed with the C57BL/6 mice (Fig. 3B), CBA/J mice infected with ∆*gra83* parasites suffered more weight loss than the controls during the acute infection (Fig. 4A), and this weight loss persisted until the end of the study. At 30 dpi, we quantified brain cyst burden and found that ∆*gra83* infection resulted in a significant increase in the cyst burden compared to GRA83^3xHA^ or GRA83c infected mice (Fig. 4B). We did not observe any gross morphological changes of the brain cysts in the mice infected with the ∆*gra83* strain (not shown). To confirm these findings, we similarly challenged C57BL/6 mice with the strains. We again observed an increase in the cyst burden of ∆*gra83* chronically infected mice, as assessed by the presence of *LDH2*-driven GFP^+^ cysts in brain sections (Fig. 4C and D). Although we cannot exclude the possibility that GRA83 plays a direct role in parasite encystment, these data suggest that the deletion of *GRA83* provides the parasite with an advantage during the acute infection, which persists and allows *T. gondii* to disseminate to the brain more efficiently, resulting in a higher cyst burden.

We also evaluated the inflammation in the cerebral tissue of the chronically infected C57BL/6 mice by histological analysis (Fig. 4E). While few inflammatory infiltrates were seen in sections of the brain parenchyma obtained from mice infected with the wild-type strain, numerous perivascular cuffings of inflammatory mononuclear cells could be observed in brain tissue sections of mice infected with ∆*gra83* strain. Infection with the *∆gra83* parasites was also associated with a severe influx of mononuclear cells to the meninges of those mice, suggestive of initial meningitis.

### GRA83 modulates IL-12/IFN-γ axis *in vitro* and *in vivo*

**Fig 5:**
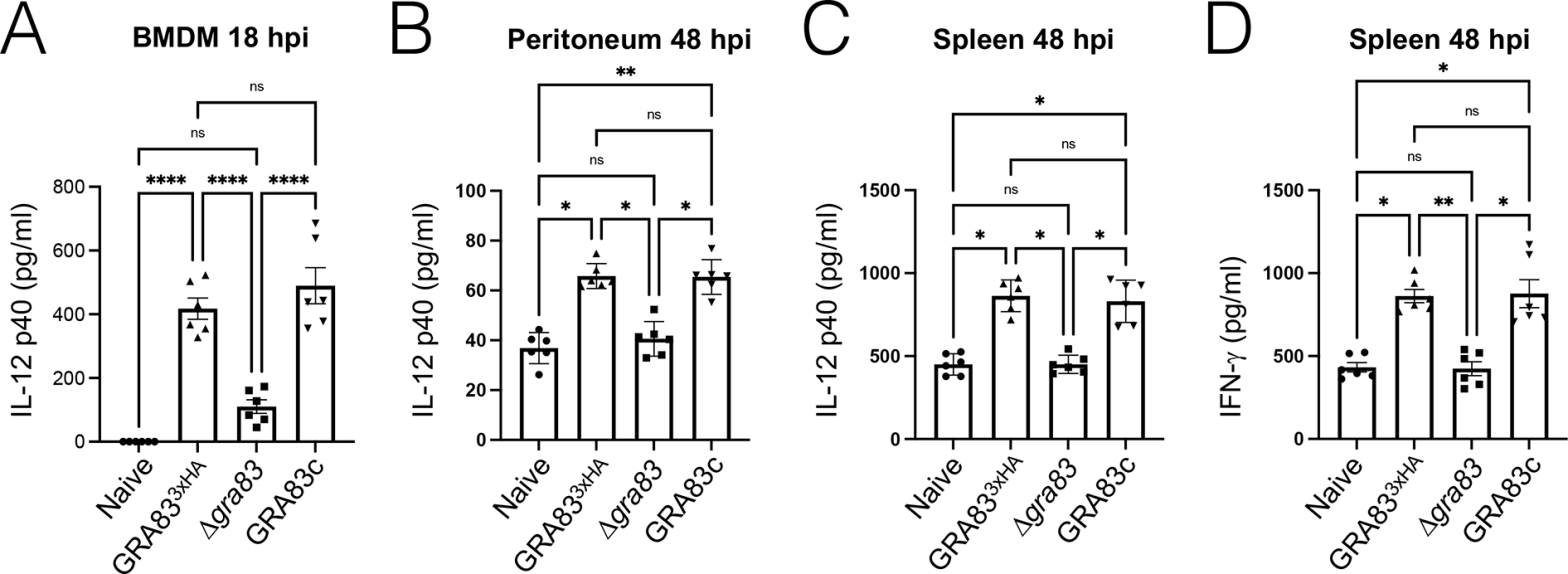
GRA83 impacts Th1 cytokine production *in vitro* and *in vivo.* (**A**) IL-12p40 production from naive and infected murine BMDMs. Cells were infected with an MOI of 0.5 of GRA83^3xHA^, ∆*gra83*, and GRA83c strains, and supernatants were collected after 18 hpi. Results are expressed as mean ± SEM of six biological replicates per group. Data were analyzed using Kruskal-Wallis one-way ANOVA test (****P < 0.001). (**B**) IL-12p40 production was measured from peritoneal exudates of C57BL/6 mice infected with 300 parasites for 48 h (*n* = 6 mice/strain). Values are expressed as mean ± SEM. Data were analyzed using Kruskal-Wallis one-way ANOVA test (*P < 0.001). (**C and D**) IL-12p40 and IFN-γ production were measured from spleen homogenates from the mice in panel B. Values are expressed as mean ± SEM. Data were analyzed using Kruskal-Wallis one-way ANOVA test (*P < 0.001).

To further explore the rapid expansion of parasites during the acute infection, we examined whether this phenomenon was due to an imbalance of the IL-12/IFN-γ axis caused by the lack of GRA83. We first measured the levels of IL-12 *in vitro*, using bone marrow-derived macrophages (BMDMs) infected with the different strains. We observed a significant decrease in the production of IL-12p40 in BMDMs after 18 h of infection with the ∆*gra83* strain*,* compared to the GRA83^3xHA^- or GRA83c-infected cells (Fig. 5A).

We additionally assessed if IL-12p40 production was altered *in vivo* by determining the cytokine concentration at the initial site of the infection (peritoneal exudates), as well as in spleen homogenates, after 48 h of infection. We found that IL-12p40 levels were again substantially reduced in both the peritoneal exudates (Fig. 5B) and spleen homogenates (Fig. 5C) of mice infected with the ∆*gra83* parasites compared to the controls. As IL-12 stimulates the production of IFN-γ, which is the crucial mediator responsible for the clearance of *T. gondii,* we also measured the concentration of this cytokine in the spleen samples. Accordingly, we found that IFN-γ production was also dramatically reduced in ∆*gra83*-infected mice compared to the controls (Fig. 5D). Together, these results suggest that the absence of GRA83 in *T. gondii* disrupts the critical pro-inflammatory IL-12/IFN-γ axis in mice, allowing uncontrolled parasite replication and severe illness for the host.

### NF-κB nuclear translocation is reduced in ∆*gra83* parasitized cells

**Fig 6:**
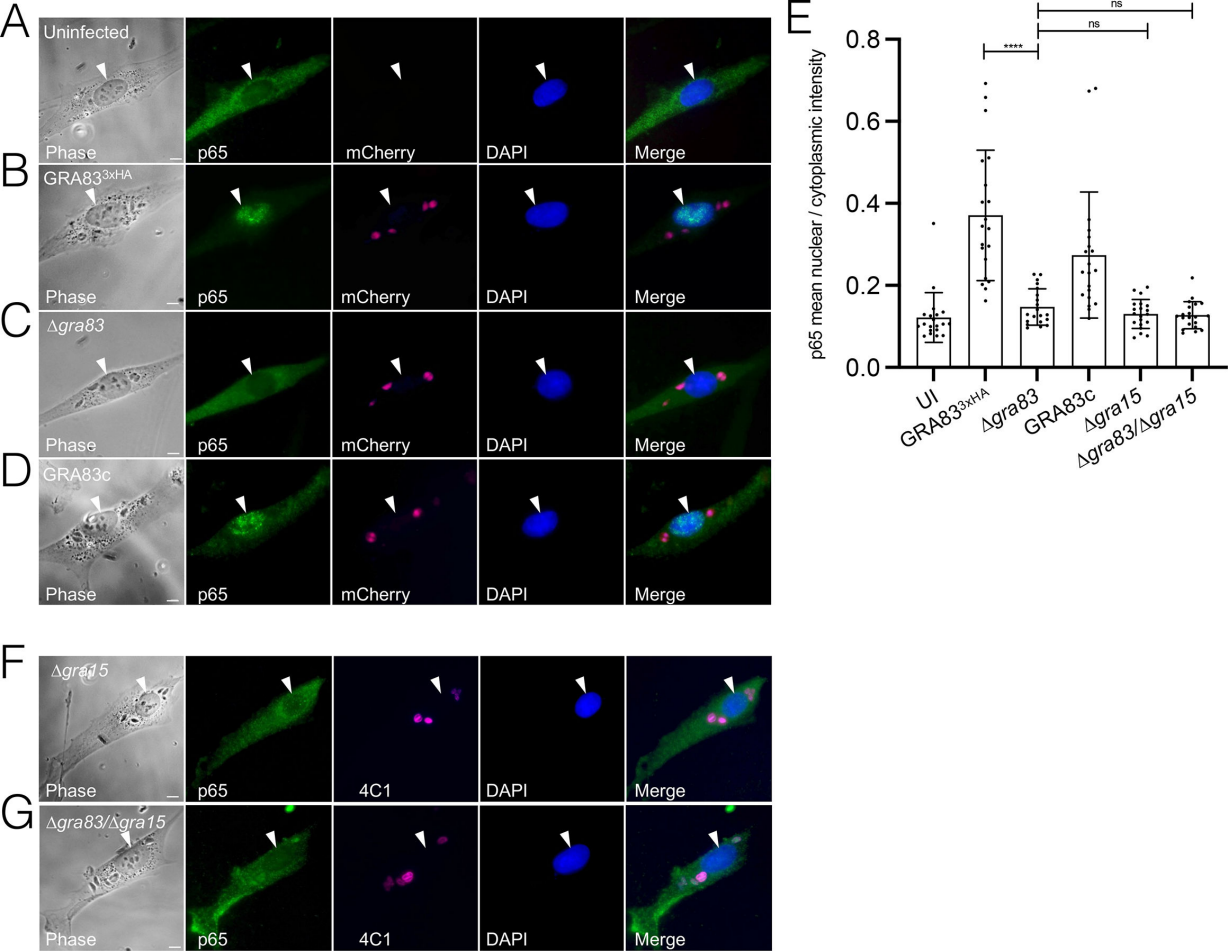
Infection with ∆*gra83* parasites results in reduced NF-κB p65 nuclear translocation in HFFs. (**A–D**) IFA of HFFs infected with *T. gondii* strains for 18 hpi, fixed, and stained with anti-p65 (green) and DAPI (blue). GRA83^3xHA^, ∆*gra83*, and GRA83c parasites expressed cytosolic mCherry (magenta) to visualize infected cells. (**F–G**) IFA of HFFs infected with ∆*gra15* or ∆*gra83*/∆*gra15* parasites at 18 hpi. Magenta: anti-parasite periphery (mAb 4C1); green: anti-p65; blue: DAPI. (**E**) The ratio of mean p65 nuclear intensity to p65 cytoplasmic intensity was quantitated in 20 HFFs for each strain. Asterisks indicate significantly higher levels of nuclear p65 compared to the uninfected cells (****P < 0.0001, ordinary one-way ANOVA test). Each arrowhead points to the host cell nucleus. Scale bars = 10 µm.

NF-κB is a central regulator of IL-12 and the subsequent IFN-γ responses to *T. gondii* infections (19, 49). To determine whether disruption of *GRA83* affects the nuclear translocation of NF-κB, we measured the nuclear fluorescence intensity of the NF-κB p65 subunit in HFFs. We observed that infection with ∆*gra83* parasites resulted in a significant decrease in p65 nuclear translocation compared to the GRA83^3xHA^ or GRA83c strains (Fig. 6A through E). GRA15 expressed by type II strains of *T. gondii* activates nuclear translocation of NF-κB in infected HFFs through its direct binding to TRAF2 and TRAF6 (31, 32). To assess the role of GRA15 in the *GRA83* knockout strain, we first epitope tagged *GRA15* in ∆*gra83* parasites, which did not show any gross changes in localization or levels of expression of the protein (Fig. S3A and B). We then generated a double knockout of *GRA83* and *GRA15* (∆*gra83/∆gra15)* to determine whether the deletion of both GRA proteins leads to an additive reduction in NF-κB translocation (Fig. S4A and B). However, we found no difference in NF-κB translocation between the ∆*gra83* and ∆*gra83/∆gra15* parasites (Fig. 6E through G). Thus, GRA83—alongside GRA15—is important for NF-κB p65 translocation to the nucleus, which leads to the production of IL-12 and, consequently, triggers IFN-γ in an *in vivo* setting. The fact that the double knockout does not appear to have an additive effect on NF-κB activation suggests that these GRAs converge to regulate this important transcription factor involved in regulating the host’s response to the infection.

### Proximity labelling identifies candidate GRA83 parasite interactors

**Fig 7:**
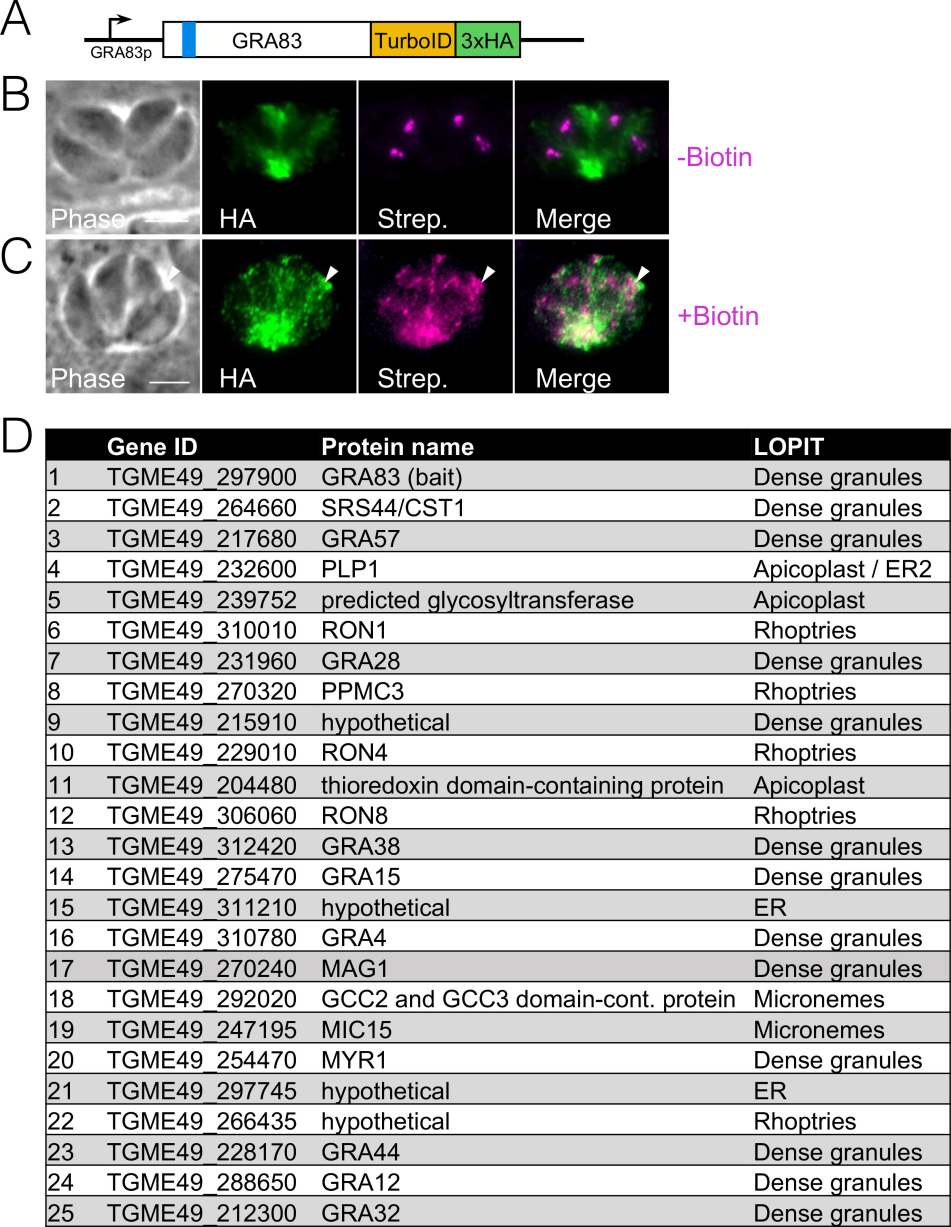
GRA83^TurboID-3xHA^ localizes to the PV and biotinylates target proteins in the PV. (**A**) Diagram showing the C-terminally tagged GRA83 with TurboID-3xHA. (**B**) IFA of GRA83^TurboID-3xHA^ without biotin supplemented to the media. The fusion protein appropriately localizes to the tachyzoite vacuole. Endogenously biotinylated proteins are detected at the apicoplast of each parasite. Magenta: streptavidin; green: anti-HA. (**C**) IFA of the GRA83^TurboID-3xHA^ expressing parasites grown for 24 h in media supplemented with biotin. Streptavidin labels the PV where the fusion protein accumulates (arrowheads). Magenta: streptavidin; green: anti-HA. Scale bar = 5 µm. (**D**) Table of the top hits from the GRA83-TurboID *in vivo* biotinylation experiment. Hits were ranked based on spectral count number and absence in the control group.

To identify candidate GRA83 interacting proteins from *T. gondii*, we used *in vivo* biotinylation with GRA83 as the bait. To do this, we endogenously tagged GRA83 with TurboID-3xHA (Fig. 7A) and showed that the fusion protein properly targeted the PV, similar to that seen for the 3xHA tagged protein (Fig. 7B). Addition of biotin to the media showed robust streptavidin labeling in the vacuole overlapping with the fusion protein, demonstrating that the fusion protein was active (Fig. 7C). We then conducted large scale proximity labeling experiments, purified the biotinylated proteins, and identified them by mass spectrometry (Fig. 7D; Fig. S5). As expected, GRA83 was the top *T. gondii* hit in the data set, likely due to self-biotinylation of the bait protein. In addition, an array of known GRA proteins involved in modulating the host during *T. gondii* infection were identified in the top 25 hits. This included GRA15, suggesting this may be a GRA83 partner in the PV. We attempted to assess GRA15/83 interaction in dual-tagged parasites by co-immunoprecipitation and western blotting, but we were unable to observe any significant interaction (not shown). Another top hit in the proximity labeling was GRA57, which is also present in the parasitophorous vacuole membrane (PVM) and has been reported to be involved with IFN-γ response (33, 50). GRA28 was also identified, which localizes to the host cell nucleus in an MYR1- and ASP5-dependent manner and facilitates parasite dissemination in hosts, and drives migration of infected macrophage (28, 51). In agreement with several of these GRAs that are secreted into the host cell, MYR1, a component of the PVM translocon, was also ranked in the top 25 hits.

## DISCUSSION

In this work, we characterize the function of the secreted *Toxoplasma* protein GRA83. GRA83 is larger than most GRAs with a predicted mass of ~83 kDa, but similar to other GRAs it lacks identifiable domains that allude to its function. The protein is largely unstructured (~40% disordered), but this is on the lower side compared to other host cell localizing GRAs (e.g., GRAs 6, 16, 24, TgIST) that range from 60 to 80% disordered (25, 29, 52, 53). We show that GRA83 is secreted into the vacuole in both tachyzoites and bradyzoites, suggesting that it functions in both lifecycle stages. While this manuscript was in preparation, another group also demonstrated that the epitope-tagged protein is secreted into the vacuole in tachyzoites (54). Together with the hyperLOPIT prediction (34), which tends to be reliable for dense granule proteins, these results confirm that GRA83 is indeed a secreted GRA protein.

We determined that the hydrophobic domain in the N-terminal portion of the protein is necessary for secretion and that the N-terminal portion of the protein, which contains this domain is sufficient for secretion. Similar N-terminal hydrophobic domains have been identified in other *T. gondii* GRA proteins including GRA6, TgNSM, and GRA15 where they may also serve as non-canonical secretory signals (30, 31, 52). As these signals are not believed to be cleaved, the hydrophobic domain may also play another role (e.g., membrane association) upon delivery to its destination. While the level of membrane association or topology of GRA83 is unknown, it may be similar to GRA6, which is believed to be tethered to the cytoplasmic face of the PVM via its N-terminal hydrophobic domain (52). Alternatively, GRA83’s function may occur entirely within the vacuole where it may reside as a membrane-associated or soluble protein. Interestingly, similar internal hydrophobic regions are also found in several secreted proteins in *Plasmodium* spp*.*, where they have recently been shown to play a direct role in ER insertion and delivery of proteins into the secretory pathway (55).

Similar to many *Toxoplasma*-secreted effector proteins that modulate host functions, GRA83 can be disrupted and the ∆*gra83* parasites grow normally in HFFs *in vitro*. This agrees with the phenotype score of 0.94 in the genome-wide CRISPR screen of type I (RH strain) parasites (56). However, infection with *∆gra83* parasites leads to a substantial increase in weight loss, parasitemia, and overall virulence in infected mice. Assessing the immune response to ∆*gra83* parasites revealed that this protein is able to induce IL-12p40 production. This triggers the assembly of critical IFN-γ dependent effector responses that will actively inhibit parasite replication, dampening the potential to cause extensive lesions in several tissues (9). The higher number of brain cysts in the chronic infection may simply be due to the higher parasitemia in the acute infection, but this may also reflect a similar role for GRA83 continuing to regulate the immune response in bradyzoites. This latter possibility is supported by the data showing that GRA83 is upregulated in bradyzoites (42). It would be interesting to test whether GRA83 driven from a tachyzoite-specific promoter still resulted in higher cyst numbers in the chronic infection.

The dampened immune response in ∆*gra83*-infected mice correlates with a reduction in NF-κB activation, which is known to control IL-12 mediated IFN-γ production (9, 30, 57). NF-κB has been shown to be primarily activated by the polymorphic GRA15 protein from the type II background strain of *T. gondii,* although GRA7 and GRA14 also have been reported to play less substantial roles in the activation (30, 58). Loss of GRA15 results in M2 macrophage polarization through decreased NF-κB activation, a reduction in IL-12 and IFN-γ production *in vivo*, and increased parasite burden, a pattern similar to what we observe for GRA83 (30, 57, 59). However, reports on the virulence of ∆*gra15* parasites appear to disagree on the increased lethality induced by the parasite in mice, a phenotype that we have confirmed in repeated experiments using ∆*gra83* parasites (30, 59 – 61). The mechanism by which GRA15 limits pathogen growth in IFN-γ stimulated human and murine fibroblasts is through direct interaction with the host ubiquitin ligases TRAF2 and TRAF6 at the vacuolar membrane, which enhances ubiquitin-mediated disruption of the vacuole (31, 32). Human monocytes also rely exclusively on GRA15 to produce the acute inflammatory IL-1β through the activation of the proteolytic inflammasome complex (62). In addition, GRA15 has been shown to activate the cGAS/STING pathway due to its localization in the host cell ER after secretion, again limiting parasite expansion and favoring chronic infection (59). While we show that GRA83 regulates NF-κB in a similar manner to that observed for GRA15, it is unlikely to directly bind TRAF2 or TRAF6, as the protein lacks the binding sites for these factors that are present in GRA15 [TRAF2: (P/S/A/T)X(Q/E)E or TRAF6: (PXEXXZ)] (32). Determining the precise mechanism by which GRA83 regulates NF-κB nuclear translocation will provide new insight into how it regulates this central control element of infection.

We were unable to observe an additive effect on the reduction of NF-κB activation from the double knockout of *GRA15* and *GRA83*. This is likely due to the convergence of both secreted proteins in the same host pathway for modulating activation. One possibility is that GRA83 and GRA15 interact and regulate each other’s function, either within the vacuole or upon its destination at the PVM. Our proximity labeling with GRA83 as bait showed that GRA15 was highly ranked in the data set, supporting this possibility. While we did not see GRA83 and GRA15 interaction by co-immunoprecipitation, this could be due to a weak interaction between the proteins or one that is unstable in detergent lysates.

While most effector proteins are aimed at interfering with the immune response to avoid pathogen clearance, it is intriguing that *T. gondii* expresses GRA15 and GRA83 that are able to limit the infection via stimulating NF-κB (30). Particularly, when the parasite also secretes effectors such as TEEGR and ROP38 that inhibit NF-κB-mediated responses (63, 64). This is likely important for regulating this pathway at different timepoints in the infection, different host cell types, or for balancing the infection in a variety of hosts. While *Neospora caninum* has a GRA83 orthologue, it lacks GRA15 as well as several other effectors that target host survival or immune evasion (e.g., GRA24, GRA28, TgIST1, TEEGR, among others) (23). *N. caninum* also does not significantly activate NF-κB (65), suggesting that GRA83 may play a role in enabling GRA15 to bind to TRAF2/6 and consequently translocate NF-κB to the nucleus, or that a second function for GRA83 in both organisms has yet to be discovered. Recently developed tools for genetic manipulation of *N. caninum* will aid in the already proven approach of comparing these pathogens using heterologous expression or knockouts of various effectors to assess how individual or groups or effectors are able to mediate infection of these pathogens in their various mammalian hosts (26, 66).

In summary, GRA83 is a secreted dense granule protein that is expressed in different life stages of *T. gondii*. It functions, by a yet unknown molecular mechanism, to regulate NF-κB translocation as well as IL-12 and IFN-γ production that help limit parasite growth *in vivo*. Together, GRA83 serves as a “pro-host” effector that counterbalances an array of “pro-parasite” effectors that collectively fine-tune the host’s immune response, likely to ensure host survival and ultimately enable transmission.

## MATERIALS AND METHODS

### Host cells and parasite culture

Parental Pru∆*ku80*∆*hxgprt* (type II, strain Prugniaud) and modified strains of *T. gondii* were maintained on confluent monolayers of HFF (BJ, ATCC, Manassas, VA, USA) and cultured as previously described (33). Constructs containing selectable markers were selected using 1 µM pyrimethamine (for dihydrofolate reductase-thymidylate synthase) or 50 µg/mL mycophenolic acid-xanthine [for hypoxanthine-xanthine-guanine phosphoribosyl transferase (HXGPRT)] (67, 68). Homologous recombination to the *UPRT* locus was negatively selected using 5 µM 5-fluorodeoxyuridine (FUDR) (46).

### Antibodies

The antibodies used in this study were mouse monoclonal antibody (mAb) HA.11 (Biolegend; 901515), rabbit polyclonal antibody (pAb) anti-HA (Invitrogen; PI715500), rat mAb anti-OLLAS (69), mouse mAb anti-4C1 (70), rabbit pAb anti-IMC6 (71), rabbit pAb anti-RON5C (72), mouse pAb anti-GRA7 (12B6) (41), mouse pAb anti-GRA14 (41), mouse pAb anti-SERCA (45), and rabbit anti-NF-κB p65 (Cell Signaling Technology; D14E12). Biotinylated proteins were detected with streptavidin Alexa Fluor 546 conjugate (Molecular Probes; S-11225).

### Epitope tagging and gene disruption

For C-terminal endogenous tagging, a pU6-Univeral plasmid containing a protospacer against the 3′ untranslated region (UTR) of the gene of interest (~150 bp downstream of the stop codon) was generated, as previously described (73). A homology-directed repair (HDR) template was PCR amplified using the ∆*ku80*-dependent LIC vector p3xHA.LIC-DHFR, smOLLAS.LIC-DHFR, or TurboID-3xHA-DHFR, which contained the epitope tag, 3′ UTR, and a selection cassette (44). The HDR templates include 40 bp of homology immediately upstream of the stop codon or 40 bp of homology within the 3′ UTR downstream of the CRISPR/Cas9 cut site. Primers that were used for the pU6-Universal plasmid, as well as the HDR template, are listed in Table S1 as P1–P10.

For gene deletions, the protospacer was designed to target the coding region of the target gene and ligated into the pU6-Universal plasmid, and prepared similar to the endogenous tagging constructs (Table S1, P11–12 and P15–16). The HDR template included 40 bp of homology immediately upstream of the start codon or 40 bp of homology downstream of the region used for homologous recombination for endogenous tagging (Table S1, P13–14 and P17–18). The HDR template was PCR amplified from a pJET vector containing the *HXGPRT* drug marker driven by the NcGRA7 promoter.

For all tagging and gene deletions, the HDR template was amplified in 400  µL, purified by phenol-chloroform extraction, ethanol precipitated, and electroporated into appropriate parasite, along with 50 µg of the pU6-Universal plasmid. Transfected parasites were allowed to invade a confluent monolayer of HFFs overnight, and the appropriate selection drug was subsequently applied. Successful tagging or deletion was confirmed by IFA, and clonal lines of tagged parasites were obtained through limiting dilution. Knockout clones were verified by PCR to examine for the absence of the gene of interest and the presence of the *HXGPRT* cassette at the predicted recombination site (Table S1, P19–25).

To generate a ∆*gra83*/∆*gra15* mutant, we first removed the *HXGPRT* cassette from the ∆*gra83* strain by transfecting in linearized NcGRA7p-pJET plasmid and a pU6-Universal plasmid containing a protospacer targeting the NcGRA7 promoter. We selected *HXGPRT*-negative clones using 6-thioxanthine (74). We next C-terminally tagged *GRA15* with the 3xHA DHFR in the parasites and subsequently deleted *GRA15* using the methods described above.

### Complementation of GRA83

GRA83-3xHA was re-introduced into the genome of the Δ*gra83 strain* in the *UPRT* locus. Primers were designed to PCR amplify the GRA83 native promoter from genomic *T. gondii* DNA of Pru∆*ku80*∆*hxgprt* (Table S1, P26–P27) and amplify the full-length, wild-type *GRA83* gene from a Pru∆*ku80*∆*hxgprt* cDNA template (Table S1, P28–29). This was cloned into the pUPRT knockout vector, resulting in the pUPRTKO-GRA83p-GRA83-3xHA. To make the GRA83c^∆72-94^ strain, primers (Table S1, P30–P31) were used to delete residues 72–94 from the pUPRTKO-GRA83p-GRA83-3xHA plasmid template using Q5 mutagenesis kit.

Each of the complementation vectors were linearized with PsiI-v2 (NEB), transfected into ∆*gra83* parasites, and selected with FUDR. Clones of each strain were selected by limiting dilution, examined by IFA (anti-HA antibody), and positive clones were designated either GRA83c or GRA83c^∆72-94^ depending on the complementation vector used.

### mCherry expressing GRA83^3xHA^, ∆*gra83*, and GRA83c *T. gondii*

One hundred micrograms of either pTub.mCherry.3xHA.UPRT or pTub.mCherry.3xHA.DHFR plasmids were linearized using PsiI-HF. GRA83^3xHA^ or ∆*gra83* parasites were transfected with the linearized pTub.mCherry.3xHA.UPRT plasmid and PU6 plasmid containing gRNA targeting the *UPRT* locus and selected with FUDR. GRA83c parasites were transfected with linearized pTub.mCherry.3xHA.DHFR and selected with pyrimethamine. After selection, parasites were cloned by limiting dilution, and cytosolic mCherry expression was confirmed by IFA.

### Immunofluorescence assay and western blot

For IFAs, glass coverslips of confluent HFFs were infected with *T. gondii* for approximately 24–34 h. The coverslips were then fixed in 3.7% formaldehyde in phosphate-buffered saline (PBS) and processed for immunofluorescence as previously described (33). Primary antibodies were detected by species-specific secondary antibodies conjugated to Alexa Fluor 488/594 (Thermo Fisher). Coverslips were mounted onto microscope slides with Vectashield (Vector Labs, Burlingame, CA, USA), or Vectashield with DAPI mounting media and the fluorescence was observed using a Zeiss Axio Imager.Z1 microscope. Images were processed with Zeiss Zen 2.3 software (Zeiss).

For the IFAs intended for determining NF-κB p65 translocation, HFFs were infected with *T. gondii* strains for 18 h, fixed, and stained, as described above. Vectashield with DAPI was used to mount coverslips onto the microscope slides. Nuclear p65 translocation was determined by calculating the ratio of the mean nuclear p65 intensity divided by the mean cytoplasmic p65 intensity, and graphs were made using Prism GraphPad. This was quantitated in 20 infected HFF cells for each strain.

For western blot**,** intracellular parasites were lysed in 1× Laemmli sample buffer with 100 mM DTT and boiled at 100°C for 10 min. Lysates were resolved by SDS-PAGE and transferred to nitrocellulose membranes overnight at 4°C. Proteins were detected with the appropriate primary antibody and corresponding secondary antibody conjugated to horseradish peroxidase. Chemiluminescence was induced using the Supersignal West Pico substrate (Pierce) and imaged on a ChemiDoc XRS+ (Bio-Rad, Hercules, CA, USA).

### *In vitro* conversion of *T. gondii* tachyzoites to bradyzoites

HFFs were grown on coverslips for 7 days and infected with GRA83^3xHA^ parasites for 3 h. The media were changed to bradyzoite “switch” media (Dulbecco's Modified Eagle Medium with 1% fetal bovine serum, 50 mM HEPES, 1% PSG, pH 8.1), and the cultures were incubated at 37°C in ambient air (0.05% CO_2_) for 3 days to induce conversion to bradyzoites, as previously described (33, 75). The coverslips were then fixed for IFA analysis as above. Dolichos biflorus agglutinin (Vector Laboratories: RL-1032) was used to stain the cyst wall of bradyzoites.

### Plaque assay

Six-well plates were seeded with confluent monolayers of HFFs and infected with approximately 250 parasites and allowed to form plaques for 8 days. The monolayers were then fixed in 100% methanol for 3 min, washed with PBS, and stained with crystal violet for visualization. The areas of at least 50 plaques per strain were measured using Zen software (Zeiss), and experiments were done in triplicates. Plaque means were compared using one-way ANOVA test. Data were graphed with GraphPad Prism (used to make all graphs).

### Mouse virulence assay

GRA83^3xHA^, ∆*gra83*, or GRA83c parasites were collected from infected NIH-3T3 monolayers and resuspended in RPMI 1640 medium (Thermo Fisher Scientific) prior to intraperitoneal injection into female C57BL/6 mice (6–8 weeks of age), with 10^3^ tachyzoites. A sample of each inoculum was stored for later confirmation by qPCR. Mice were monitored for symptoms of infection, weight loss, and survival for 20 days.

### Determination of parasite burden

The parasite burden was determined in C57BL/6 mice infected with GRA83^3xHA^, ∆*gra83*, and GRA83c tachyzoites. Peritoneal cells were submitted to a real-time qPCR using SYBR green detection system (PowerUp SYBR Green Master Mix, Thermo Fisher). Genomic DNA was extracted by salting out. DNA concentrations were quantified by spectrophotometer (260/280 ratio; Nanodrop Lite, Thermo Scientific) and adjusted to 40 ng/µL with sterile DNase-free water. The reaction to determine parasite load was performed in a Real-time PCR thermal cycler (StepOne Plus, Thermo Scientific, USA) and parasite genomic DNA was quantified by interpolation from a standard curve with known amounts of DNA extracted from *T. gondii* tachyzoites that were included in each analysis. Primers used to examine the repetitive 529 bp DNA fragment in *T. gondii* are included in Table S1 as P32–33.

### Histological analysis

Lung and brain samples of *T. gondii* infected C57BL/6 mice were collected and fixed in 10% buffered formalin at room temperature for 24 h and stored in 70% alcohol until the paraffin inclusion process. After inclusion, the organs were sliced (5 mm thick) and deposited on microscopic slides, subsequently stained with hematoxylin and eosin for the evaluation of inflammatory infiltrates and tissue damage as previously described (76). The stained sections were photographed using an automated slide scanner microscope (Aperio ScanScope AT, Leica). The histopathological profile of the tissues was blindly determined by a veterinary pathologist.

### Mouse brain cyst quantitation

Intracellular GRA83^3xHA^, ∆*gra83*, and GRA83c parasites were mechanically liberated from infected HFF monolayers and resuspended in Opti-MEM prior to intraperitoneal injection into groups of female CBA/J mice each. Four hundred tachyzoites per mouse of each strain were injected. Injected parasites were confirmed to be live and viable parasites by plaque assays with HFF monolayers. The mice were monitored for symptoms of infection and weight loss 30 days after injection and then sacrificed. For the chronic infection model, these mice were sacrificed at the conclusion of the experiment at 30 days post-infection. Euthanasia was done per AVMA guidelines—using slow (20–30% per minute) displacement of chamber air with compressed CO_2_. This is followed by a confirmatory method of cervical dislocation. Mouse brains were collected and *LDH2-*GFP^+^ tissue cysts were quantified, as previously described (33).

For the C57BL/6 strain, mice were infected with 300 tachyzoites and monitored for 32 days for clinical signs. Mice were then euthanized and had the brain removed and divided in half: one hemisphere was homogenized and examined by phase microscopy for tissue cysts; the other hemisphere was placed in an embedding medium (Tissue-Tek O.C.T. Compound, Sakura Finetek) and quickly frozen in liquid nitrogen. The tissues were submitted to cryosectioning and counterstained with DAPI. The slides were read in a fluorescent microscope (EvosFL, Thermo), and tissue cysts were identified by the expression of *LDH2*-GFP^+^.

### Cytokine measurements

C57BL/6 BMDMs were differentiated and stimulated as previously described (77). Briefly, the cells were seeded (2 × 10^5^ per well) in 96-well plates and left to adhere overnight at 37°C in 5% CO_2_. Cells were infected with freshly lysed GRA83^3xHA^, ∆*gra83,* and GRA83c tachyzoites at an MOI of 0.5, and supernatants (200 µL) were collected at 18 h after infection and stored at −80°C. For *in vivo* assays, C57BL/6 mice were euthanized after 48 h of infection, and the peritoneal cavity was washed with 1 mL of PBS, spun at 400 × *g* for 10 min to pellet the cells. The supernatant was collected and stored at −80°C. Spleens were also collected at 48 h, mechanically disrupted by a tissue homogenizer (Ika) in 1 mL of PBS with protease inhibitor cocktail (Roche), and spun at 10,000 × *g* for 10 min to pellet debris. Supernatants were collected and stored at −80°C. IL-12p40 and IFN-γ levels were determined using commercially available enzyme-linked immunosorbent assay kits (BD Biosciences) according to the manufacturer’s instructions.

### Affinity capture of biotinylated proteins

HFF monolayers were infected with parasites expressing the GRA83^TurboID-3xHA^ or the parental Pru∆*ku80*∆*hxgprt* strain. Two replicates of each were used. Upon infection, 150 µM biotin was supplemented in the media for 30 h. Intracellular parasites were collected, washed in PBS, and lysed in radioimmunoprecipitation assay (RIPA) buffer [50 mM Tris (pH 7.5), 150 mM NaCl, 0.1% SDS, 0.5% sodium deoxycholate, 1% NP-40] supplemented with Complete Protease Inhibitor cocktail (Roche) for 30 min on ice. Lysates were centrifuged for 15 min at 14,000 × *g* to pellet insoluble material and the supernatant was incubated with Streptavidin Plus UltraLink Resin (Pierce) at 4°C overnight under gentle agitation. Beads were collected and washed five times in RIPA buffer, followed by three washes in 8 M urea buffer (50 mM Tris-HCl pH 7.4, 150 mM NaCl). Ten percent of each sample was boiled in Laemmli sample buffer, and eluted proteins were analyzed by western blotting by streptavidin-HRP, while the remainder was used for mass spectrometry.

### Mass spectrometry of biotinylated proteins

The proteins bound to streptavidin beads were reduced and alkylated via sequential 20-min incubations of 5 mM TCEP and 10 mM iodoacetamide at room temperature in the dark while being mixed at 1,200 rpm in an Eppendorf thermomixer. Proteins were then digested by the addition of 0.1 µg Lys-C (FUJIFILM Wako Pure Chemical Corporation, 125-05061) and 0.8 µg of Trypsin (Thermo Scientific, 90057) while shaking at 37°C overnight. The digestions were quenched via the addition of formic acid to a final concentration of 5% by volume. Each sample was desalted via C18 tips (Thermo Scientific, 87784) and then resuspended in 15 µL of 5% formic acid before analysis by liquid chromatography-tandem mass spectrometry.

Peptide samples were separated on a 75 µM ID, 25 cm C18 column packed with 1.9 µM C18 particles (Dr. Maisch GmbH) using a 140 min gradient of increasing acetonitrile and eluted directly into a Thermo Orbitrap Fusion Lumos instrument where MS/MS spectra were acquired by data dependent acquisition. Data analysis was performed using the ProLuCID and DTASelect2 algorithms (78 – 80) as implemented in the Integrated Proteomics Pipeline—IP2 (Integrated Proteomics Applications, Inc., San Diego, CA, USA) against the *T. gondii* ME49 reference proteome from ToxoDB and the human reference proteome from Uniprot. Protein and peptide identifications were filtered using DTASelect and required a minimum of two unique peptides per protein and a spectrum-level false positive rate of less than 1% as estimated by a decoy database strategy. Candidates were ranked by spectral counts comparing GRA83^TurboID^ vs control samples. Significance Analysis of INTeractome (SAINT) on the hits was done using the SAINTexpress_v3.6.3 program.

## SUPPLEMENTAL MATERIAL

Fig. S1 msphere.00263-23-s0001.tif Alphafold provides a low confidence structure of GRA83.

Fig. S2 msphere.00263-23-s0002.tif The N terminus of GRA83 is sufficient for secretion to the PV.

Fig. S3 msphere.00263-23-s0003.tif Deletion of *GRA83* does not impact GRA15 expression levels or localization.

Fig. S4 msphere.00263-23-s0004.tif Verification of GRA15 disruption by western blot and PCR analyses.

Fig. S5 msphere.00263-23-s0005.xlsx Hits identified by mass spectrometry of GRA83-TurboID whole cell lysates.

Table S1 msphere.00263-23-s0006.tif Oligonucleotide primers.

ASM does not own the copyrights to Supplemental Material that may be linked to, or accessed through, an article. The authors have granted ASM a non-exclusive, world-wide license to publish the Supplemental Material files. Please contact the corresponding author directly for reuse.
