# Supplementary figures and images for: The Toxoplasma gondii effector GRA83 modulates the host’s innate immune response to regulate parasite infection
Source: mSphere. 2023 Sep 28;8(5):e00263-23. doi: 10.1128/msphere.00263-23 (PMC10597413; doi:10.1128/msphere.00263-23)

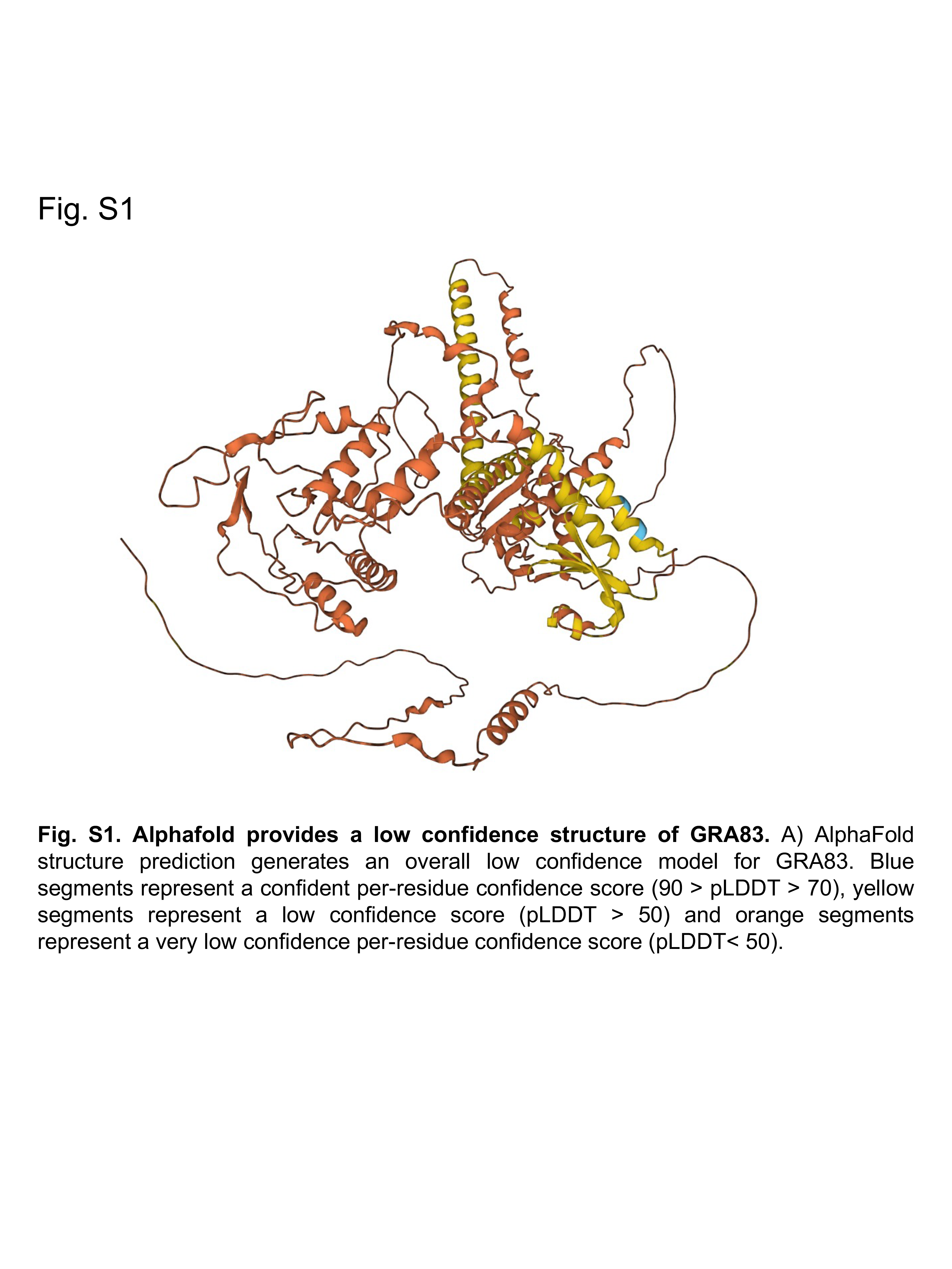

Supplement: Fig. S1 — Alphafold provides a low confidence structure of GRA83. [file msphere.00263-23-s0001.tif]

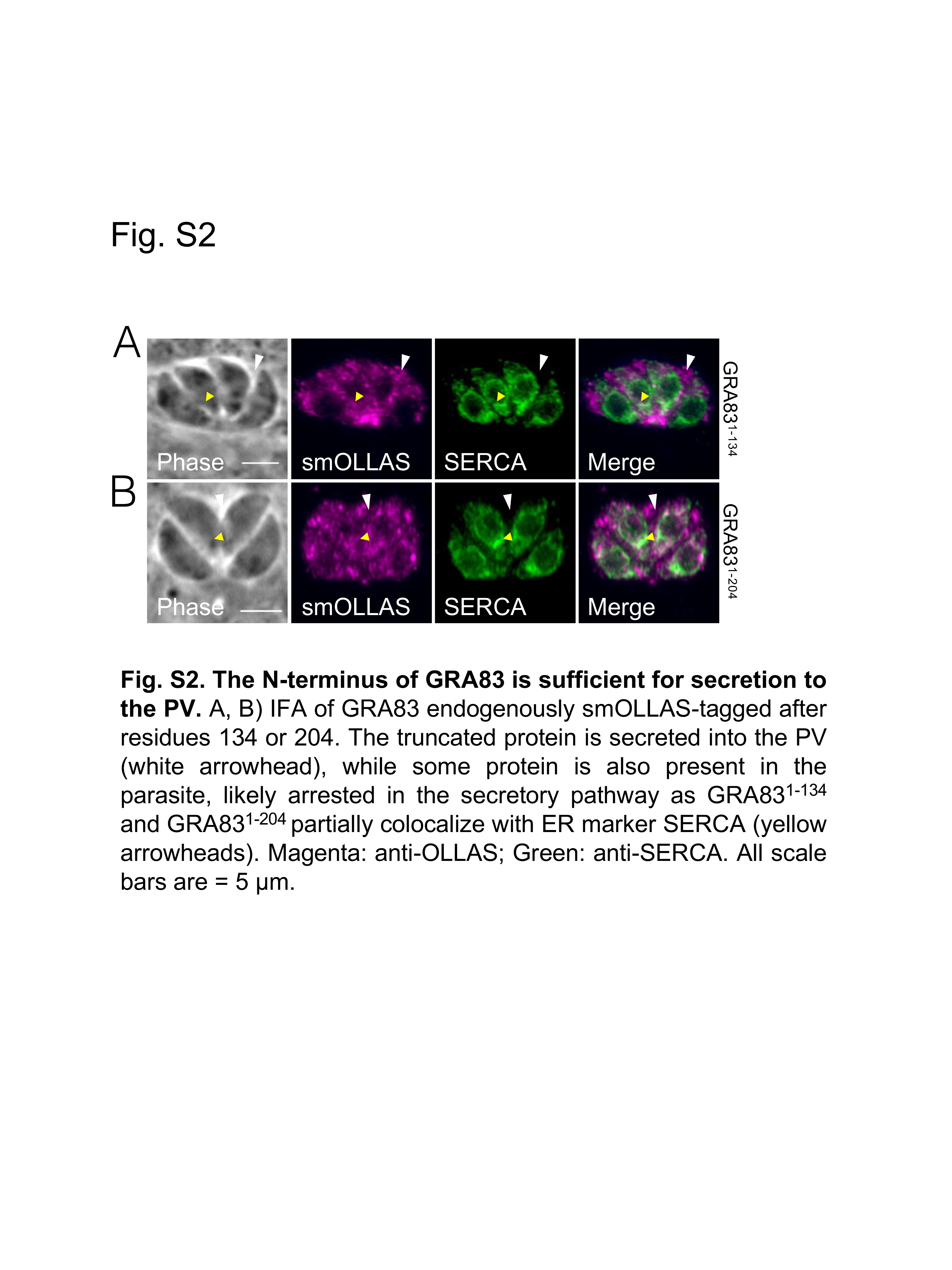

Supplement: Fig. S2 — The N terminus of GRA83 is sufficient for secretion to the PV. [file msphere.00263-23-s0002.tif]

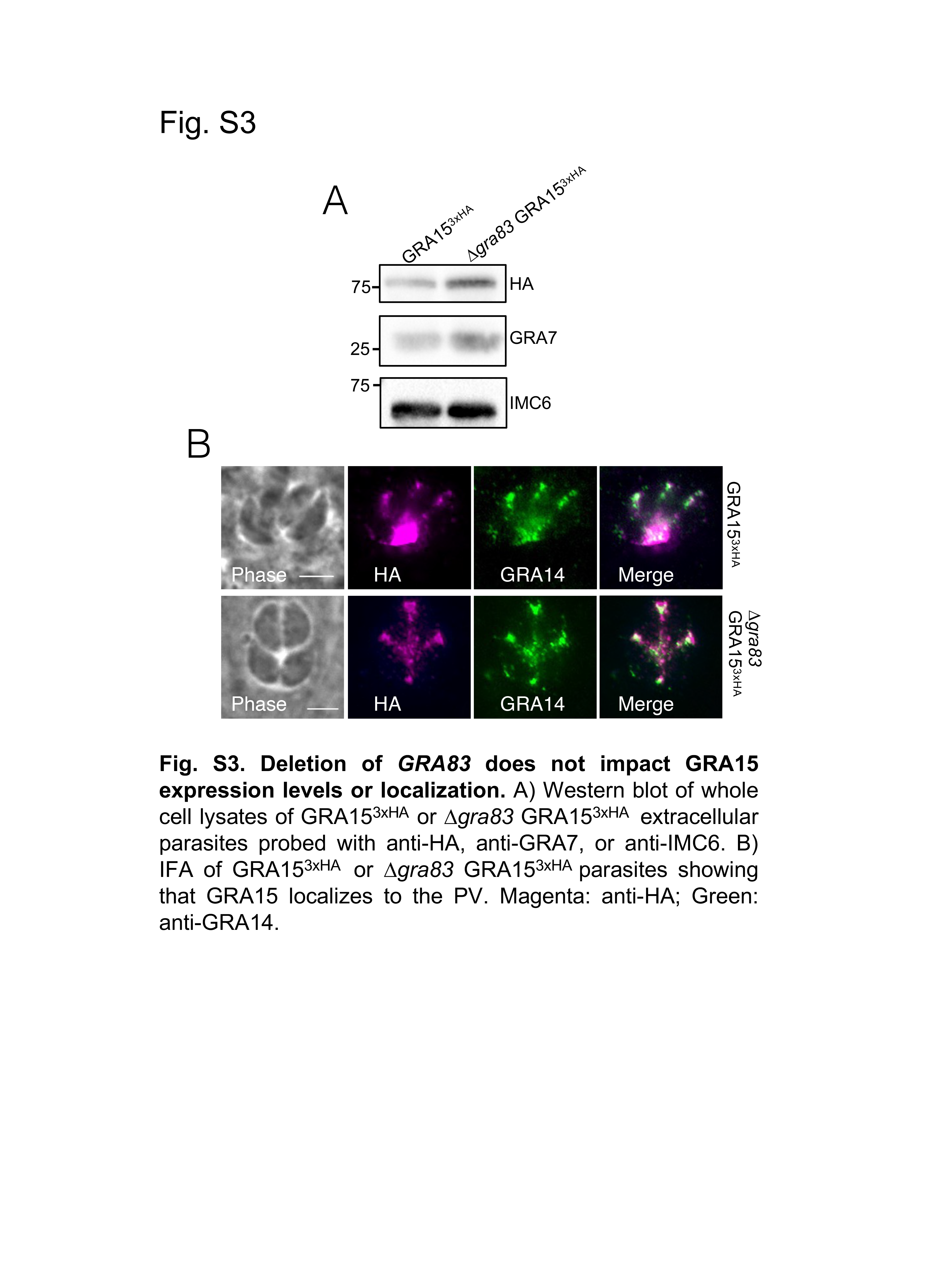

Supplement: Fig. S3 — Deletion of GRA83 does not impact GRA15 expression levels or localization. [file msphere.00263-23-s0003.tif]

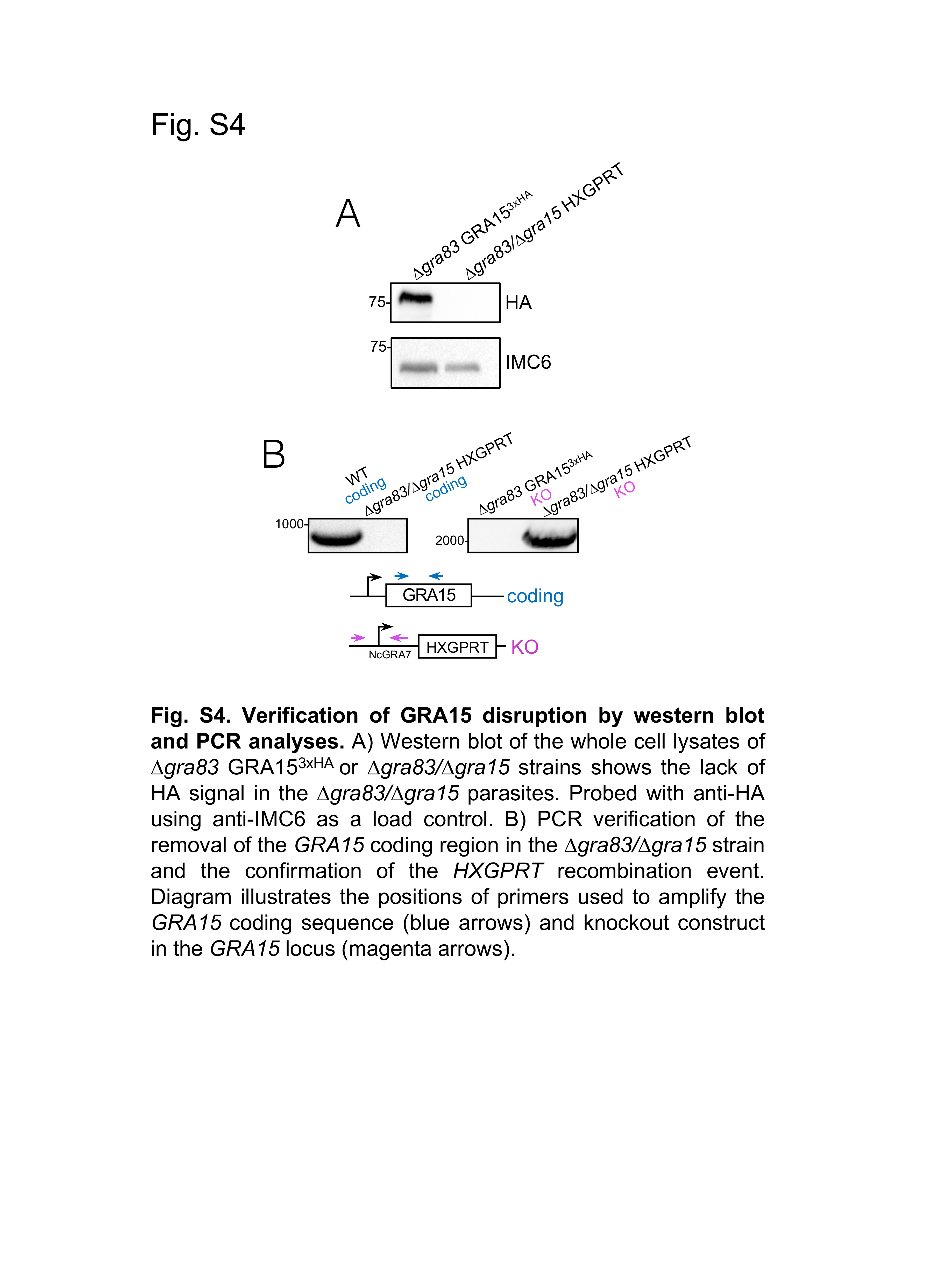

Supplement: Fig. S4 — Verification of GRA15 disruption by western blot and PCR analyses. [file msphere.00263-23-s0004.tif]

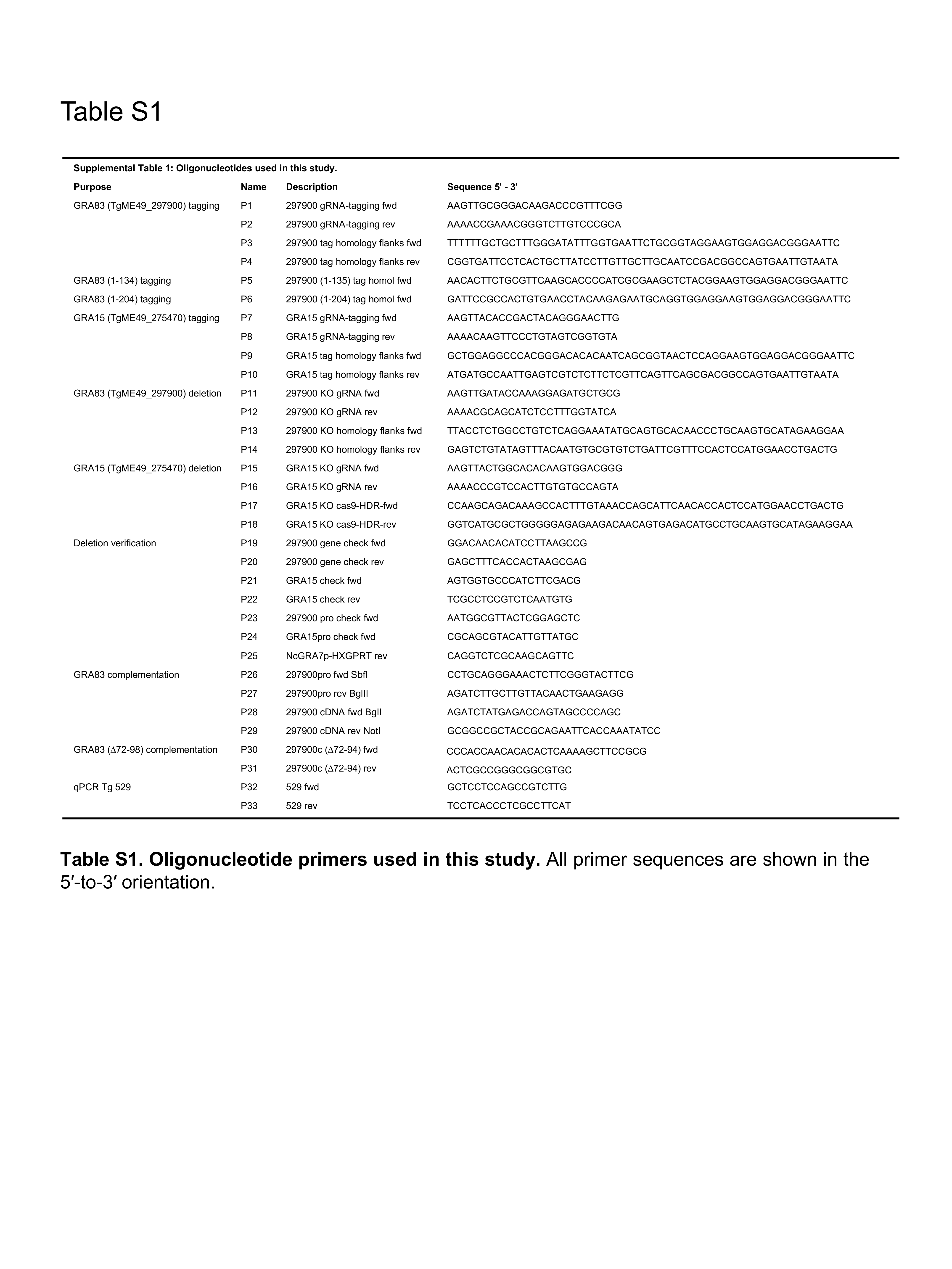

Supplement: Table S1 — Oligonucleotide primers. [file msphere.00263-23-s0006.tif]
